# Persistent Effect of Gait Exercise Assist Robot Training on Gait Ability and Lower Limb Function of Patients With Subacute Stroke: A Matched Case–Control Study With Three-Dimensional Gait Analysis

**DOI:** 10.3389/fnbot.2020.00042

**Published:** 2020-07-24

**Authors:** Yiji Wang, Masahiko Mukaino, Satoshi Hirano, Hiroki Tanikawa, Junya Yamada, Kei Ohtsuka, Takuma Ii, Eiichi Saitoh, Yohei Otaka

**Affiliations:** ^1^Department of Rehabilitation Medicine I, School of Medicine, Fujita Health University, Toyoake, Japan; ^2^Department of Spinal Cord Injury Rehabilitation, China Rehabilitation Research Center, Capital Medical University, Beijing, China; ^3^School of Rehabilitation Medicine, Capital Medical University, Beijing, China; ^4^Faculty of Rehabilitation, School of Health Sciences, Fujita Health University, Toyoake, Japan; ^5^Department of Rehabilitation, Fujita Health University Hospital, Toyoake, Japan

**Keywords:** rehabilitation, robot-assisted gait training, stroke, gait ability, lower limb function

## Abstract

**Introduction:**

Gait exercise assist robot (GEAR), a gait rehabilitation robot developed for poststroke gait disorder, has been shown to improve walking speed and to improve the poststroke gait pattern. However, the persistence of its beneficial effect has not been clarified. In this matched case–control study, we assessed the durability of the effectiveness of GEAR training in patients with subacute stroke on the basis of clinical evaluation and three-dimensional (3D) gait analysis.

**Methods:**

Gait data of 10 patients who underwent GEAR intervention program and 10 patients matched for age, height, sex, affected side, type of stroke, and initial gait ability who underwent conventional therapy were extracted from database. The outcome measures were walk score of Functional Independence Measure (FIM-walk), Stroke Impairment Assessment Set total lower limb motor function score (SIAS-L/E), and 3D gait analysis data (spatiotemporal factors and abnormal gait patter indices) at three time points: baseline, at the end of intervention, and within 1 week before discharge.

**Results:**

In the GEAR group, the FIM-walk score, SIAS-L/E score, cadence, and single stance time of paretic side at discharge were significantly higher than those at post-training (*p* < 0.05), whereas the stance time and double support time of the unaffected side, knee extensor thrust, insufficient knee flexion, and external rotated hip of the affected side were significantly lower (*p* < 005). However, no significant differences in these respects were observed in the control group between the corresponding evaluation time points.

**Conclusion:**

The results indicated significant improvement in the GEAR group after the training period, with respect to both clinical parameters and the gait pattern indices. This improvement was not evident in the control group after the training period. The results possibly support the effectiveness of GEAR training in conferring persistently efficient gait patterns in patients with poststroke gait disorder. Further studies should investigate the long-term effects of GEAR training in a larger sample.

## Introduction

Various assisted gait exercise robots have been developed and used in clinical practice. Previous studies have shown the effectiveness of several robots, such as Lokomat and Gait Trainer, in improving the walking ability of stroke patients when used in combination with routine physiotherapy compared with physiotherapy alone (Pohl et al., 2007; Schwartz et al., 2009; Morone et al., 2011).

The gait exercise assist robot (GEAR) is a type of gait rehabilitation robot developed to support gait practice of patients with poststroke severe hemiplegia (Hirano et al., 2017). Most of the previously reported gait exercise robots provide gait training of symmetrical gait pattern with the robotic devices worn on both lower limbs (Hesse et al., 1999; Colombo et al., 2000; Van Der Kooij et al., 2006). However, patients with severe hemiplegia need to walk with an asymmetrical gait pattern because of compensatory motion; therefore, the aim of gait practice is not to acquire the normal, symmetrical gait but to establish the best efficient gait pattern within the limitations of motor impairment. The GEAR is a stationary, one-leg GEAR designed to assist only the hemiplegic lower limb. The device is highly adjustable and incorporates various feedback mechanisms. The adjustability in the level of assistance enables the patients to deal with the changes in the severity of motor impairment during the subacute phase of stroke; in addition, the feedback facilitates motor learning of the patients. Previous studies have shown that the use of GEAR in addition to conventional physiotherapy helps achieve markedly improved walking ability and gait parameters compared with those in control patients who undergo physiotherapy alone (Katoh et al., 2019; Tomida et al., 2019). Notably, a kinematic analysis study with three-dimensional (3D) motion analysis system revealed the effectiveness of GEAR training in reducing abnormal patterns, as assessed by spatiotemporal and kinematic indices (Katoh et al., 2019).

However, many of the abnormal gait patterns are attributable to the compensatory movements in response to poststroke motor impairment and may be optimal for the level of hemiplegia. Therefore, it is possible that the improvement in the abnormal gait patterns merely reflect the temporal changes due to the GEAR-induced forced gait pattern; in effect, these changes may be negated after a certain period of time after completion of GEAR training. No previous studies have evaluated the durability of the improved gait patterns conferred by GEAR training using 3D gait analysis. In this context, it should be meaningful to evaluate the persistence of the effects of GEAR intervention on walking ability.

The purpose of this retrospective study was to investigate the persistent effect in gait ability with 3D gait analysis after the completion of GEAR training by comparing with that of strictly matched control subjects who did not undergo GEAR training.

## Materials and Methods

### Participants

Individuals who had already completed GEAR program and who been evaluated on three occasions, that is, before the first training session (baseline), immediately after the last training session (post-training), and within 1 week before discharge (retention), were identified from the gait analysis database. Patients who did not receive GEAR training were identified from the same database as control group and matched forage (within ± 3 years), height (within ± 4 cm), sex, affected side, type of stroke, and initial Stroke Impairment Assessment Set total lower limb motor function score (SIAS-L/E) (within ± 1 point) (Chino et al., 1994). Then, gait data pertaining to three time points for each control were selected from their gait records by matching the time frame of gait analysis in the GEAR group. Inclusion criteria for the study were as follows: (1) unilateral stroke (hemorrhagic or ischemic); (2) first stroke; (3) ability to understand and follow the instructions of the researcher; and (4) ability to walk independently with cane or orthosis. The exclusion criterion was as follows: presence of orthopedic disease or severe cardiopulmonary disease that limits gait ability. Finally, 10 patients as GEAR group and 10 individuals as control group were matched.

This study was approved by the institutional review board. Written informed consent was obtained from all subjects.

### Protocol of Gait Training Using the Gait Exercise Assist Robot

**FIGURE 1:**
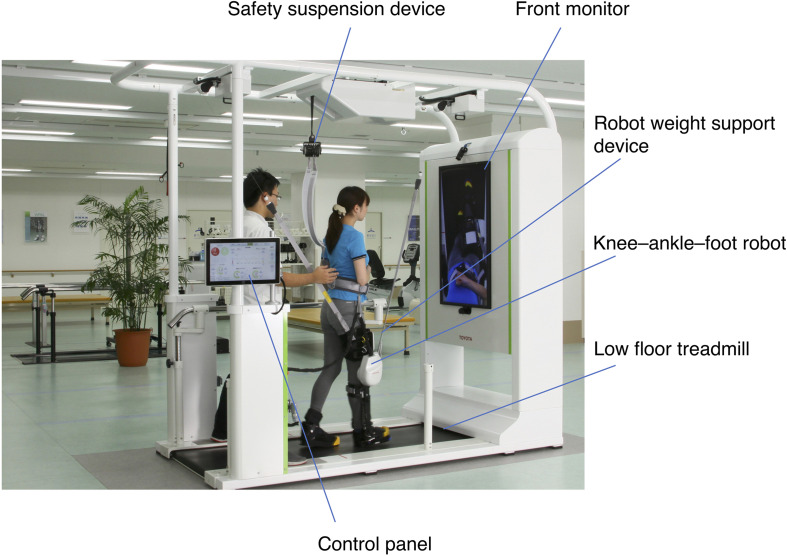
Structure of the gait exercise assist robot (GEAR). The system consists of a knee–ankle–foot robot, a low floor treadmill, a safety suspension device, a robot weight support device, a front monitor for feedback to the patient, and a control panel. The control panel can be turned to the opposite direction so that the therapists standing behind the patient can control the level of assistance and receive the feedback during the training.

The GEAR has been developed by Fujita Health University and Toyota Motor Corporation for the purpose of supporting gait training of poststroke hemiplegic patients (Hirano et al., 2017). The GEAR includes a knee–ankle–foot robot, a low floor treadmill, a safety suspension device (can be used as a body weight support device), a robot weight support device, a monitor for patient use, and a control panel (Figure 1). The GEAR incorporates a variety of feedback and task difficulty settings. For example, as a feedback for patients, the monitor at the front can display either the full-length image (mirror image) or the foot image. As acoustic feedback, the device can be set to emit a sound of success when the weight on the hemiplegic side exceeds the set value and a sound of failure when the knee gives way. The task settings system includes various assist controls such as knee extension assist and amount of body weight support.

The GEAR group received gait training using the GEAR for 40 min a day including putting-on and taking-off time, 5–7 days a week. Training on the GEAR was conducted by a certificated therapist who also decided the content of training. Gait training using the GEAR was continued until the patient could walk overground stably for more than 10 m using a cane and an ankle–foot orthosis (AFO) under supervision. During the period of GEAR training, patients in the GEAR group also received physical therapy and occupational therapy for no more than 180 min a day. Patients in the control group received the same therapy except for GEAR training.

### Outcome Measures

Spatiotemporal factors and the degree of 10 abnormal gait patterns (Table 1; Itoh et al., 2012; Tanikawa et al., 2016; Hishikawa et al., 2018), which were calculated using a 3D motion capture system with force plate measurement (KinemaTracer, Kissei Comtec Co., Ltd., Matsumoto, Japan) at post-training and before discharge, were used to evaluate the persistence of the effect of gait training on gait patterns. Three-dimensional gait analysis at baseline was not conducted because it is difficult for subjects with severe hemiplegia to complete the 3D analysis on the treadmill at that time.

**TABLE 1:** Calculation of index values representing the severity of abnormal gait.

Abnormal gait	Calculation of index values
Circumduction gait	The difference in distance between the lateralmost *X* coordinate of the ankle joint marker in 25–75% of the swing phase and the medial-most *X* coordinate in 25–75% of the stance phase, corrected by lower limb length.^1^
Hip hiking	The difference between the maximum value of the *Z* coordinate of the hip joint marker during the swing phase and the *Z* coordinate of the contralateral hip joint marker at the same time, corrected for the mean left-right difference of the *Z* coordinate during the double support phase.
Retropulsion of the hip	The average distance between the *X* coordinate of the ankle joint and the *X* coordinate of the toe in the swing phase, corrected by lower limb length.^1^
Excessive hip external rotation	The average distance between the *X* coordinate of the ankle joint and the *X* coordinate of the toe in the swing phase, corrected by foot length.^2^
Knee extensor thrust	The difference between the maximum *Y* coordinate velocity of the knee in the single stance phase and treadmill gait speed.
Flexed-knee gait	The maximum knee extension angle during single stance phase.
Insufficient knee flexion during the swing phase	The percentage difference in the maximum knee flexion angle during swing phase between the patient and healthy subjects.
Forefoot contact	The difference in distance between the *Z* coordinate of the ankle joint marker and the *Z* coordinate of the toe marker at initial contact, minus the difference in distance between the *Z* coordinates of the ankle joint marker and toe marker during standing.
Medial whip	The difference in distance between the medial-most *X* coordinate of the ankle joint marker during 25–75% of the stance phase and the lateralmost *X* coordinate during 75–100% of the stance phase, corrected by foot length.^2^
Excessive lateral shift of the trunk over the unaffected side	The average distance between (1) the lateralmost *X* coordinate of the midpoint between the bilateral acromions in the part of the double stance phase in which the affected leg is located behind the unaffected leg and the swing phase of affected leg and (2) the average *X* coordinate of the midpoint between the bilateral ankle joints in the part of the double stance phase in which the affected leg is located behind the unaffected leg, corrected by lower limb length.^1^

**X*, *Y*, and *Z* coordinates indicate lateromedial, anteroposterior, and vertical directions, respectively. ^1^Corrected by lower limb length: the index value was expressed as a percent of the *Z* coordinate of the hip joint in the standing position. ^2^Corrected by foot length: the index value was expressed as a percent of the distance in the *Y* coordinate between the ankle joint and the toe in the standing position.*

The clinical outcome measures included comfortable gait velocity measured by 10-m walk test (10 MWT) (Lord and Rochester, 2005), walk score of Functional Independence Measure (FIM-walk) (Keith et al., 1987), and SIAS-L/E score at the baseline, post-training, and before discharge.

**FIGURE 2:**
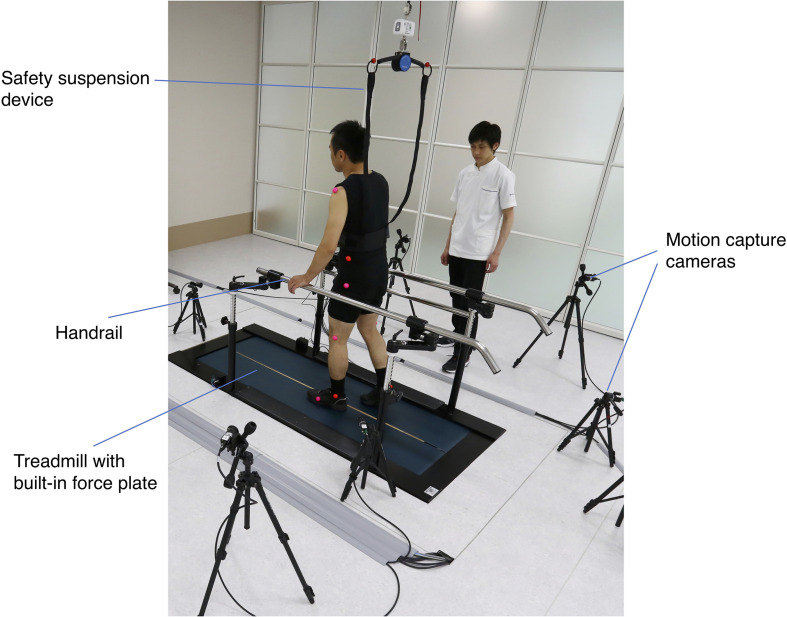
Setting of three-dimensional (3D) gait analysis. The measurement was conducted on the treadmill with built-in force plate. The limb movement of the patient during walking was captured by eight optical cameras placed around the treadmill. A safety suspension device and a handrail were provided to prevent falls during the measurement.

The setting of gait analysis is presented in Figure 2. Twelve colored markers placed on bilateral shoulder, pelvis, hip, knee, ankle, and the fifth metatarsal head were used (Mukaino et al., 2018). The comfortable gait speed of subjects was subjectively assessed based on the 10 MWT. After achievement of a steady state on the treadmill, data were collected for 20 s; data for at least five complete gait cycles were collected for each subject. The index values for spatiotemporal factors and degree of abnormal gait patterns were determined automatically by the system on the basis of the data of force plate and eight synchronized charge-coupled device camera sets.

### Statistical Analysis

All statistical analyses were performed using SPSS version 18.0 for Windows (SPSS Inc., Chicago, IL, United States). The normality of distribution of quantitative variables was assessed using the single sample Kolmogorov–Smirnov test. Paired *t*-test as the parametric test and the Wilcoxon signed-rank test as the nonparametric test were used to assess between-group differences. Chi-squared test was used for qualitative variables. One-way repeated measures analysis of variance was performed to examine within-group differences over the three measurement periods. *p-*values < 0.05 were considered indicative of statistical significance.

## Results

The characteristics of subjects are presented in Table 2. No significant differences were observed between the GEAR group and control group with respect to demographic characteristics (e.g., age), affected side, or the initial clinical characteristics (e.g., SIAS-L/E and FIM-walk score).

**TABLE 2:** Demographic and clinical characteristics of the study population.

	GEAR group	Control group	
	(*n* = 10)	(*n* = 10)	*p-*Value
Age (years)	60 ± 10	59 ± 11	0.307
Height (cm)	162.3 ± 7.1	161.8 ± 7.7	0.742
Gender (male/female)	5/5	5/5	0.999
Affected side (left/right)	4/6	4/6	0.999
Lesion type (ischemic/hemorrhagic)	1/9	1/9	0.999
Initial SIAS-L/E	1.0 ± 1.3	1.2 ± 1.7	0.443
SIAS-L/E at T0	3.8 ± 1.3	4.2 ± 2.2	0.494
FIM-W at T0	2.2 ± 0.8	2.4 ± 0.8	0.555
Velocity at T0 (km/h)	0.31 ± 0.2	0.23 ± 0.1	0.153
Handrail (+/−) at T0	10/0	10/0	0.999
KAFO/AFO at T0	8/2	5/5	0.170
Duration of T0–T1 (weeks)	4 ± 3	5 ± 3	0.213
Duration of T1–T2 (weeks)	5 ± 2	5 ± 2	0.726
Duration of T0–T2 (weeks)	9 ± 3	10 ± 4	0.462

*GEAR, gait exercise assist robot; SIAS-L/E, Stroke Impairment Assessment Set total lower limb motor function score; FIM-W, walk score of Functional Independence Measure; KAFO: knee–ankle–foot orthosis; AFO, ankle–foot orthosis; T0, the date of gait assessment before the first GEAR training session (baseline); T1, the date of gait assessment immediately after the last GEAR training session (post-training); T2, the date of gait assessment within 1 week before discharge (retention).*

### Clinical Assessment

**FIGURE 3:**
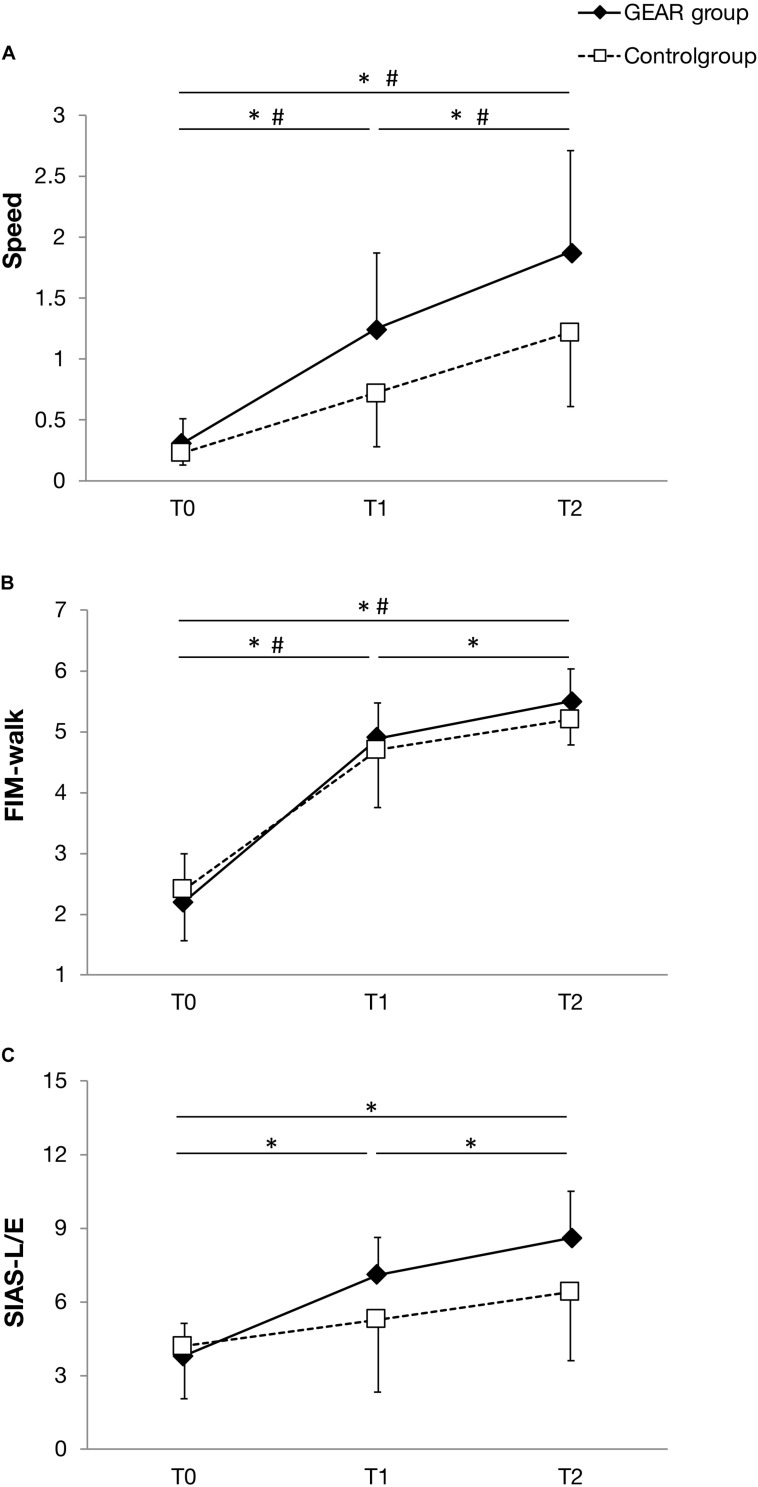
Line plot depicting the mean and standard deviation of the parameters at T0, the date of gait assessment before the first gait exercise assist robot (GEAR) training session (baseline); T1, the date of gait assessment immediately after the last GEAR training session (post-training); T2, the date of gait assessment within 1 week before discharge (retention) in the GEAR and control groups. **(A)** Speed; **(B)** FIM-walk; and **(C)** SIAS-L/E. * indicates significant difference between time points in the GEAR group. # indicates significant difference between time points in the control group. FIM-walk, walk score of Functional Independence Measure; SIAS-L/E, Stroke Impairment Assessment Set total lower limb motor function score.

In the GEAR group, the FIM-walk score and SIAS-L/E score at discharge were significantly greater than those at the post-training time point (*p* < 005); SIAS-L/E score at discharge and post-training was significantly greater than that at the pre-training time point (*p* < 005). However, no significant differences in this respect were observed in the control group between the corresponding time points (Figure 3).

### Spatiotemporal Indices

In the GEAR group, the cadence and the percentage of nonparetic swing time (paretic single stance time) at discharge were significantly larger than the corresponding post-training values (*p* < 005); in addition, the percentage of nonparetic stance time and double support time at discharge was significantly lower than that at post-training (*p* < 005). No significant differences in these respects were observed in the control group between the corresponding time points (Table 3).

**TABLE 3:** Spatiotemporal indices at the three study time points.

Variables	GEAR group						Control group					
	Affected side			Unaffected side			Affected side			Unaffected side		
	T1	T2	P	T1	T2	P	T1	T2	P	T1	T2	P
Cadence (steps/min)	73.2 ± 16.1	87.3 ± 19.4*	0.023				59.1 ± 14.5	68.5 ± 18.2	0.174			
Stride length (cm)	55.1 ± 20.9	67.0 ± 26.1*	0.028				44.2 ± 20.9	55.9 ± 18.2*	0.013			
Step length (cm)	29.9 ± 9.9	35.6 ± 12.8*	0.021	25.1 ± 11.8	31.1 ± 14.5	0.064	23.0 ± 10.3	29.2 ± 9.6*	0.034	20.9 ± 11.6	26.4 ± 9.6	0.057
Stance time (%)	68.2 ± 6.1	66.7 ± 6.0	0.294	79.2 ± 5.1	75.5 ± 5.9*	0.003	72.1 ± 3.6	67.5 ± 4.8*	0.001	82.4 ± 4.2	80.8 ± 5.8	0.266
Swing time (%)	31.8 ± 6.1	33.3 ± 6.0	0.294	20.8 ± 5.1	24.5 ± 5.9*	0.003	27.9 ± 3.6	32.5 ± 4.8*	0.001	17.6 ± 4.2	19.2 ± 5.8	0.266
Double support time (%)	24.6 ± 7.0	23.2 ± 6.5	0.312	22.9 ± 3.9	18.9 ± 4.2*	0.001	30.4 ± 8.6	27.0 ± 9.9*	0.043	24.1 ± 6.5	21.4 ± 4.1	0.083

GEAR, gait exercise assist robot; T1, the date of gait assessment immediately after the last GEAR training session (post-training); T2, the date of gait assessment within 1 week before discharge (retention). *indicates significant difference between T2 and T1 (*p* < 0.05).

### Gait Abnormality Indices

The degree of abnormal gait pattern is indicated by the deviation value, which is directly provided by the Kinema Tracer system.

$$\mathrm{The}\,\mathrm{deviation}\,\mathrm{value}\mathrm{The}\,\mathrm{deviation}\,\mathrm{value}\;=\frac{10\times \left({\mathrm{subject}}^{\prime }\,s\,\mathrm{value}-\mathrm{mean}\,\mathrm{normal}\,\mathrm{value}\right)}{\mathrm{standard}\,\mathrm{deviation}}+50.$$

The normal range for deviation value of each gait pattern is 30–70, which was defined as the mean ± 2SD values obtained in healthy subjects (Itoh et al., 2012; Tanikawa et al., 2016; Hishikawa et al., 2018). Therefore, presence of deviation value of gait pattern indices of more than 70 or less than 30 was regarded as gait abnormality in this study. In the GEAR group, indices for knee extensor thrust and insufficient knee flexion of the affected side at discharge were significantly lower than those of the post-training level (*p* < 005); no significant difference in this respect was observed in the control group (Table 4).

**TABLE 4:** Abnormal gait patterns on the affected side over time.

Measure	GEAR group			Control group		
	T1	T2	P	T1	T2	P
Forefoot contact	64.0 ± 10.0	67.7 ± 6.2	0.311	69.2 ± 8.3	63.5 ± 12.1	0.097
Knee extensor thrust	86.6 ± 9.3	80.2 ± 11.0*	0.005	88.3 ± 13.5	86.6 ± 12.8	0.777
Retropulsion of the hip	72.5 ± 15.7	75.5 ± 19.2	0.619	90.6 ± 16.7	87.1 ± 12.9	0.531
Flexed knee gait	44.2 ± 11.1	51.1 ± 17.4*	0.046	34.8 ± 7.0	44.3 ± 8.1*	0.005
Insufficient knee flexion	114.8 ± 17.5	100.2 ± 18.8*	0.002	130.6 ± 19.1	119.7 ± 11.1	0.103
Medial whip	69.8 ± 25.4	77.0 ± 23.2	0.337	66.1 ± 23.6	97.8 ± 57.0	0.159
Circumduction gait	72.8 ± 20.8	67.6 ± 25.8	0.515	89.2 ± 48.9	87.7 ± 42.8	0.903
Hip hiking	71.9 ± 11.5	69.6 ± 11.0	0.502	87.5 ± 19.5	81.1 ± 12.1	0.107
Lateral shift of the trunk	94.1 ± 36.8	91.1 ± 26.8	0.692	95.8 ± 20.7	103.6 ± 31.4	0.502
External rotated hip	85.3 ± 18.6	75.4 ± 19.1*	0.005	71.4 ± 18.3	73.9 ± 18.9	0.330

GEAR, gait exercise assist robot; T1, the date of gait assessment immediately after the last GEAR training session (post-training); T2, the date of gait assessment within 1 week before discharge (retention). *indicates significant difference between T2 and T1 (*p* < 005).

## Discussion

In this study, GEAR helped maintain the improved ambulatory ability and locomotor recovery in patients after completion of the intervention. Our findings are consistent with those of previous studies and demonstrate the long-term effectiveness of repetitive gait training in the subacute phase of stroke (Pohl et al., 2007; Ng et al., 2008). This is the first study that showed the persistent effect of GEAR training on abnormal gait patterns using 3D gait analysis.

Evaluation of higher functional level performance provided some interesting results. The FIM-walk test showed persistent improvement in motor skills and functional gait in patients with severe hemiplegia even some weeks after the completion of GEAR training. This indicates a long-lasting motor recovery on the basis of functional relearning induced by early, repetitive, and intense gait-focused training in the early stage after stroke (Da Cunha et al., 2002; Pohl et al., 2007). By providing appropriate levels of assist for swing and stance, GEAR can provide repetitive, task-oriented, extensive walking training from an early stage (Hirano et al., 2017).

Gait exercise assist robot facilitated durable improvement in cadence after the completion of training, which is consistent with previous studies that used treadmill with partial body weight support systems (BWSs) or robotic-aided gait training (RAGT) (Molteni et al., 2015; Gama et al., 2017). GEAR can help patients to study how to launch and swing out the paralyzed hip quickly and adequately, which can help increase cadence during the training. Specifically, patients can easily build on and strengthen the memory to swing out the hip owing to the repetitive and large amount of walking training provided by GEAR. Therefore, patients are able to maintain their swinging-out movement after GEAR training. In the GEAR group, the paretic single stance time at discharge was longer and the nonparetic double support time at discharge was shorter compared with those at the post-training level; this reflects the greater stability of the affected lower limb during weight bearing, which is a sign of improved dynamic gait stability and greater confidence in shifting body weight (Wallard et al., 2015; Yeung et al., 2018). Collectively, these modifications brought about improved postural stability and control of dynamic balance during follow-up, which may also be related to the motor learning imparted by the GEAR.

The improvement in gait performance in the GEAR group at discharge indicates that patients tend to acquire better gait patterns through the course of GEAR gait training and tend to maintain the gait pattern after training. This result may be related to the extensive repetition of gait pattern, which is effective in establishing the robust pattern (Wallard et al., 2015). The GEAR enables repetition of similar gait patterns from early stage to the end stage of the rehabilitation. Usually, poststroke patients exhibit gradual improvement in motor impairment (Jorgensen et al., 1995). At the beginning of the usual gait training, the practice starts with the gait pattern optimized according to the severity of motor impairment at that time (Eng and Tang, 2007). As the motor impairment improves over time, there is a change in the adapted gait pattern, which necessitates modification of the practice. In contrast, the GEAR training starts with the assistance by the robot, which enables the patient to walk with less compensatory gait patterns. As the GEAR allows adjustment in the level of assistance, the patients can practice the similar gait pattern for several weeks, with different levels of assistance optimized for the severity of paresis at a particular time. The assistance will be reduced alongside the improvement in hemiplegia; however, the gait pattern does not change significantly throughout the robot training period. This might render the gait pattern more robust and thus enhance improvement in gait ability after the GEAR training period.

In addition, these abnormal patterns are believed to be related to the weakness of lower limb muscles (Moore et al., 1993; Moseley et al., 1993; Hishikawa et al., 2018); thus, the reduction in these parameters may also be related to improved paresis recovery.

In the present study, subjects in the GEAR group showed continual gains in SIAS score even after the completion of GEAR training. During exercise using GEAR, minimizing the assist level according to the patient’s capability helps increase the opportunities to exercise lower limb during walking; previous studies have shown that active robotic training may hasten the recovery of lower limb function, facilitate motor learning-related neuroplasticity (Krishnan et al., 2012, 2013), and help reduce compensatory movements (Katoh et al., 2019). After the GEAR training, the reduced compensatory movements may increase the chances to exercise lower limb during walking. For example, hip hiking is one of the major compensatory movements in hemiparetic gait (Stanhope et al., 2014). The extent of hip hiking shows a negative correlation with the extent of paretic limb shortening by knee flexion during the swing phase (Matsuda et al., 2017); if the paretic limb shortening is poor, hip hiking should be greater to ensure toe clearance. Acquisition of gait pattern with less compensation requires patients to move their paretic joints; this increases the opportunity to exercise lower limb during walking, even after the GEAR training. This idea may be supported by the present results as the knee extensor thrust and insufficient knee flexion, which are both related to the control ability of knee movements, were improved after the GEAR training. Further studies are required for more in-depth characterization of the mechanisms underlying the persistent effect of GEAR.

### Limitation

This study has several limitations. Firstly, this was a retrospective study with a small number of patients. However, we matched the controls with respect to several potential confounding factors that may affect gait comparison, which ensured homogeneity between groups. Secondly, the observation period after the end of intervention ranged from 2 to 8 weeks. However, it was matched one to one to maximize comparability. Nonetheless, it may be difficult to confirm the accurate durability of effects because of the absence of same follow-up time; therefore, due caution should be exercised while interpreting our results. However, it provided an opportunity to observe the temporal changes in gait ability and provided a reference to determine the time points for assessment of long-term effects of GEAR in a future study.

## Conclusion

In this study, the persistent effect of early, repetitive, and intensive gait-focused training provided by GEAR was examined in comparison with the strictly matched control using 3D gait analysis. Despite the sample size, our results support the superiority of repetitive GEAR training over usual training in terms of durability of the improvement in gait parameters. Our results call for further studies to clarify the effectiveness of GEAR training in a larger sample.

## Data Availability Statement

The datasets generated for this study are available from the corresponding author on reasonable request.

## Ethics Statement

The studies involving human participants were reviewed and approved by the Fujita Health University ethics committee. The patients/participants provided their written informed consent to participate in this study. Written informed consent was obtained from the individuals for the publication of any potentially identifiable images or data included in this article.

## Author Contributions

YW and MM designed the study, developed the protocol, and carried out the data analysis. YW, MM, and YO wrote the manuscript. YW, HT, JY, TI, and KO participated in clinical data collection. YW, MM, SH, HT, KO, ES, and YO participated in interpretation of the data. ES and YO supervised the whole process of the study. All authors read and approved the final manuscript.

## Conflict of Interest

ES received collaborative research funding from Toyota Motor Corporation, which developed the robot discussed in this article. The remaining authors declare that the research was conducted in the absence of any commercial or financial relationships that could be construed as a potential conflict of interest.

## Abbreviations

AFO: ankle–foot orthosis
BWS: body weight support system
FIM-walk: walk score of Functional Independence Measure
GEAR: gait exercise assist robot
KAFO: knee–ankle–foot orthosis
RAGT: robotic-aided gait training
SIAS-L/E: Stroke Impairment Assessment Set total lower limb motor function score
10MWT: 10-m walk test.
